# Dominant Retinitis Pigmentosa, p.Gly56Arg Mutation in *NR2E3*: Phenotype in a Large Cohort of 24 Cases

**DOI:** 10.1371/journal.pone.0149473

**Published:** 2016-02-24

**Authors:** Fiona Blanco-Kelly, María García Hoyos, Miguel Angel Lopez Martinez, Maria Isabel Lopez-Molina, Rosa Riveiro-Alvarez, Patricia Fernandez-San Jose, Almudena Avila-Fernandez, Marta Corton, Jose M. Millan, Blanca García Sandoval, Carmen Ayuso

**Affiliations:** 1 Genetic´s Department, Instituto de Investigación Sanitaria-Fundación Jimenez Diaz University Hospital (IIS-FJD, UAM), Madrid, Spain; 2 Center of Biomedical Network Research on Rare Diseases (CIBERER), ISCIII, Madrid, Spain; 3 Instituto de Medicina Genómica (IMEGEN), Valencia, Spain; 4 Department of Ophthalmology, Instituto de Investigación Sanitaria-Fundación Jiménez Díaz University Hospital (IIS-FJD, UAM), Madrid, Spain; 5 Grupo de Investigación en Biomedicina Molecular, Celular y Genómica, Instituto de Investigación Sanitaria La Fe (IIS-La Fe), Valencia, Spain; 6 Unidad de Genética y Diagnóstico Prenatal, Hospital Universitario y Politécnico La Fe, Valencia, Spain; Hadassah-Hebrew University Medical Center, ISRAEL

## Abstract

**Importance:**

This research is the single largest *NR2E3* genotype-phenotype correlation study performed to date in autosomal dominant Retinitis Pigmentosa.

**Objective:**

The aim of this study is to analyse the frequency of the p.Gly56Arg mutation in *NR2E3* for the largest cohort of autosomal dominant Retinitis Pigmentosa patients to date and its associated phenotype.

**Patients and Methods:**

A cohort of 201 unrelated Spanish families affected by autosomal dominant Retinitis Pigmentosa. The p.Gly56Arg mutation in the *NR2E3* (NM_014249.2) gene was analysed in 201 families. In the 24 cases where the mutation had been detected, a haplotype analysis linked to the p.Gly56Arg families was performed, using four extragenic polymorphic markers D15S967, D15S1050, D15S204 and D15S188. Phenotype study included presence and age of onset of night blindness, visual field loss and cataracts; and an ophthalmoscopic examination after pupillary dilation and electroretinogram for the 24 cases.

**Results:**

Seven of the 201 analyzed families were positive for the p.Gly56Arg, leading to a prevalence of 3.5%. Clinical data were available for 24 subjects. Night blindness was the first noticeable symptom (mean 15.9 years). Visual field loss onset was variable (23.3 ± 11.9 years). Loss of visual acuity appeared late in the disease´s evolution. Most of the patients with cataracts (50%) presented it from the third decade of life. Fundus changes showed inter and intrafamiliar variability, but most of the patients showed typical RP changes and it was common to find macular affectation (47.4%). Electroretinogram was impaired from the beginning of the disease. Two families shared a common haplotype. Additionally, all patients shared a 104Kb region between D15S1050 and the *NR2E3* gene.

**Conclusions:**

This study highlights the importance of p.Gly56Arg in the *NR2E3* gene as a common mutation associated with adRP, and provides new clues to its phenotype, which can allow for a better clinical management and genetic counselling of patients and their families.

## Introduction

Retinitis Pigmentosa (RP, MIM# 268000), with a prevalence of approximately one in 4000 [1], is the most common form of inherited retinopathy. RP is a group of clinically and genetically heterogeneous retinal degenerative diseases. Clinically it is characterized by progressive loss of photoreceptors and pigment deposits predominantly in the peripheral retina, and by a relative sparing of the central retina. The diagnostic criteria for RP were established by Marmor [2–5]. To date, sixty-nine genes have been associated with non-syndromic RP (http://www.sph.uth.tmc.edu/RetNet/, data accessed 30/12/2015) and all modes of inheritance have been described in this disease: autosomal dominant, autosomal recessive, X linked, mitochondrial and, in rare cases, digenic [6].

In Spain, autosomal dominant form of RP (adRP) represents approximately 15% of Spanish RP families [7, 8]. The large number of genes involved in adRP disease complicates genetic analysis of these patients. To date, 23 genes (and one mapped locus) have been associated with adRP (http://www.sph.uth.tmc.edu/RetNet/, data accessed 30/12/2015). One of these 23 genes is the *NR2E3* gene, which contains eight exons that expand a genomic sequence of around 7.7 kilobases (kb). The open reading frame of this gene encodes for a retinal nuclear receptor protein that acts as a transcriptional regulator, activating rod-specific genes in concert with other transcriptional factors (CRX and NRL), as well as repressing the transcription of cone-specific genes in differentiating rod photoreceptors [9].

Most of the mutations in this gene have been associated with autosomal recessive retinitis pigmentosa (arRP) with variable phenotypes (enhanced S-cone sensitivity syndrome -ESCS- [10, 11], Goldmann-Favre syndrome -GFS- [12], and clumped pigmentary retinal degeneration -CPRD-) [13–16]. However, one mutation (p.Gly56Arg) in the first zinc-finger of the DNA binding domain of the *NR2E3* gene has been found in adRP patients [17] associated with RP phenotype (progressive rod degeneration and ulterior cone affectation) [18]. This mutation accounts for approximately 1–2% of North American and 3.4% of European adRP patients [17–19], and, until present time, is the only mutation found in the *NR2E3* gene responsible for adRP [17–21].

The aim of this study is to analyse the frequency of the p.Gly56Arg mutation in the *NR2E3* gene in our cohort of adRP patients and to determine the associated phenotype.

## Patients and Methods

### Patients

The adRP diagnosis was based on pedigree data and ophthalmologic examination. Our patients were classified as affected by RP according to the following clinical criteria: night blindness (NB), progressive loss of peripheral vision (mid peripheral scotoma or ring scotoma), fundus compatible with RP [3, 4] (ophthalmoscopic examination after pupillary dilation), and pathologic electroretinogram (ERG) showing a marked reduction in rod or rod and cone signal (full-field electroretinogram according to the standards of the International Society for Clinical Electrophysiology of Vision: http://www.iscev.org) [22]. Autosomal dominant inheritance was considered according to previously established criteria [7, 8].

The severity of visual acuity loss was classified following the WHO criteria (normal vision ≥0.4, moderate low vision <0.4 –>0.1, severe low vision ≤0.1 - ≥0.05, and profound vision loss and blindness <0.05)

Written informed consent was obtained from all individuals included in the study and research protocols were approved by the Ethics committee of the University Hospital Fundación Jiménez Díaz in accordance with the tenets of the Declaration of Helsinki and their reviews.

### Screening for *NR2E3* autosomal dominant mutation

DNA was extracted from peripheral blood samples and collected in EDTA tubes using an automated DNA extractor according to manufacturer instructions (model BioRobot EZ1; Qiagen, Hilden, Germany).

The p.Gly56Arg mutation in the *NR2E3* gene (NM_014249.2) was analysed in a total of 201 unrelated adRP families. The analysis was performed by direct sequencing, as previously reported [19], or by adRP genotyping Asper Ophthalmics microarray (Asper Biotech [19], http://www.asperbio.com/asper-ophthalmics/autosomal-dominant-retinitis-pigmentosa-ad-rp/autosomal-dominant-retinitis-pigmentosa-targeted-mutation-analysis, versions from February 2008 to July 2014).

Among 201 families, 60 had been studied previously, showing a negative result, using the first version of the adRP genotyping microarray, which did not include the p.Gly56Arg mutation [23]. The remaining 141 families underwent an updated version of the adRP genotyping microarray which included the p.Gly56Arg mutation in the *NR2E3* gene. Direct sequencing was used to analyse the 60 previously studied families, to confirm the results obtained with the genotyping microarray and to segregate the disease causative mutation p.Gly56Arg in the *NR2E3* gene in the families.

### Haplotype Analysis

Haplotype analysis was performed using four extragenic polymorphic markers (*NR2E3* genomic position according to Human Genome Assembly GRCh37, Chr15: 72,084,977–72,110,600) strongly linked to this locus: D15S967, D15S1050, D15S204, and D15S188. For the genotyping process, PCR products were electrophoresed in an ABI Prism 3130 Genetic Analyzer and analyzed with the GeneMapper v3.5 software package (Applied Biosystems).

**Fig 1:**
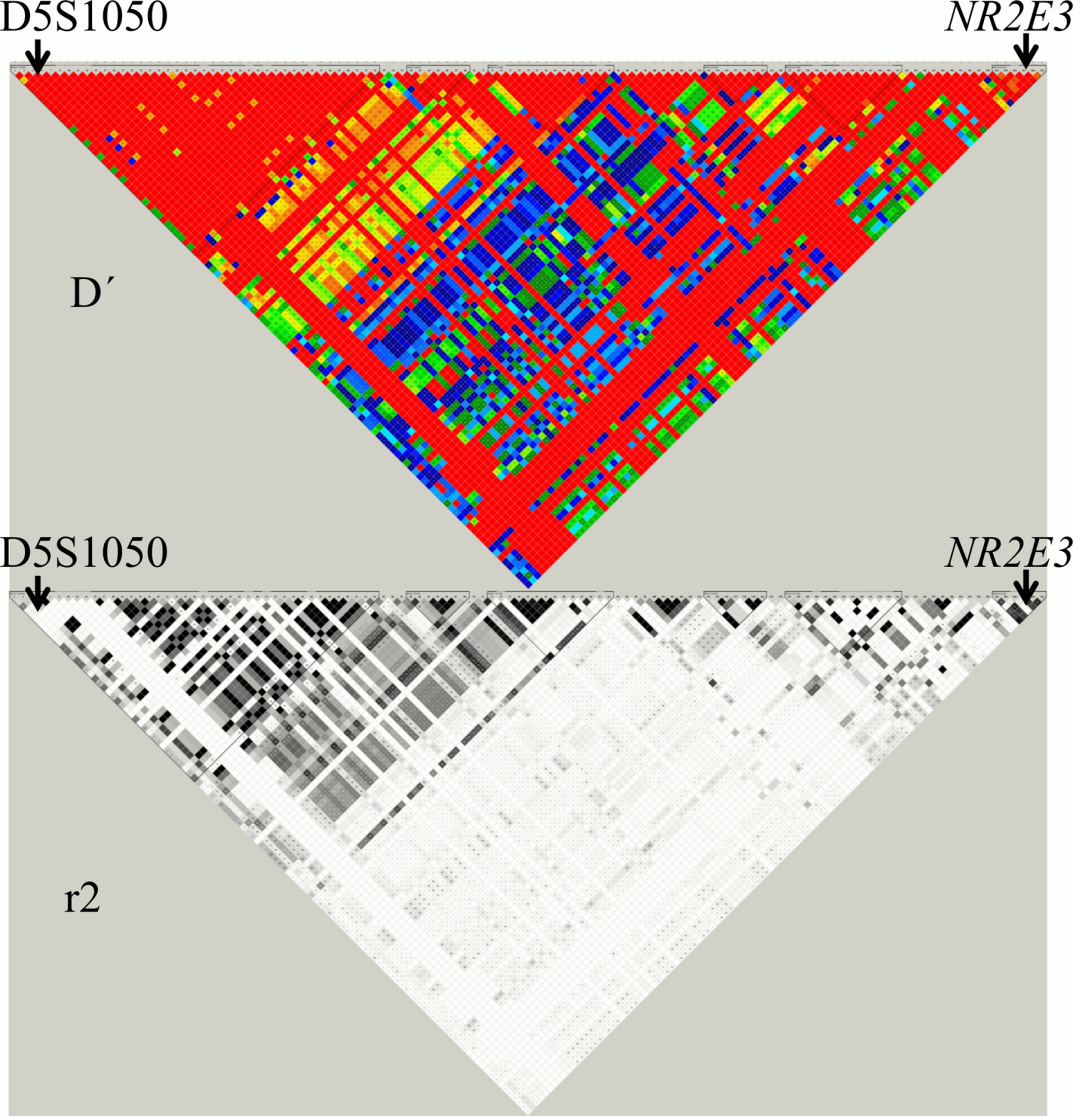
Linkage disequilibrium for region Chr15: 71,980,969–72,110,600 of the Human Genome Assembly GRCh37 (Hapmap/Haploview 4.0 software: http://www.hapmap.org/). Colour image: D´ (red: D´ = 1, the lower the D´ the further away from red). Black & white image: r^2^ (black: r^2^, the lower the r^2^ the further away from black).

An *in silico* analysis was performed using the Hapmap/Haploview 4.0 software (http://www.hapmap.org/) to establish the linkage disequilibrium blocks (D´ and r2 parameters) in the genomic region between D15S1050 and the *NR2E3* gene (Fig 1).

## Results

Seven out of 201 adRP studied families were positive for the p.Gly56Arg mutation in the *NR2E3* gene (five of them identified by direct sequencing of the mutation and two by the adRP genotyping microarray).

The detection of the dominant *NR2E3* mutation in seven families, gives us a frequency of 3.5% (7/201) in our adRP studied cohort. All families were of Spanish origin except RP-1650 which was from Venezuela (both grandparents from mother´s side–disease origin–were Venezuelan).

### Phenotypic characteristics of *NR2E3* mutation

Clinical data were available for 24 subjects from the seven *NR2E3* mutated adRP families (Table 1).

**Table 1:** Clinical data of 24 affected cases presenting the p.Gly56Arg mutation in the *NR2E3* gene.

Individual	Family	Year of birth	Age at diagnostic (years)	Age of NB onset (years)	Age of VF loss onset (years)	Age of cataracts onset (years)*	Age at VA measurement	VA	VA classification^a^	Age at VF measurement	VF	VF classification^b^	Age at evaluation of fundus	Fundus	ERG / Age (yr)	Other
I:2	RP-0030	1915	-	9yr	9yr	Yes at 74yr	78yr	perception of light	3	-	-	-	78yr	typical + pigment deposit in macula	NR / 78yr	-
II:2	RP-0030	1938	-	5yr	-	Yes (age unknown)	6yr	-		71yr	<10° ct	3	71yr	typical (few spicules) + white yellowish spots in posterior pole	NR / 71yr	-
II:5	RP-0030	1943	15yr	8yr	34yr	36yr	46yr & 66yr	BE = 0.2 → hand movement	1→2	48yr	10° ct	2	66yr	typical + macular atrophy and hypopigmented lesions	NR / 66yr	-
III:2	RP-0030	1968	-	7yr	15yr	32yr	40yr	BE = 0.8	0	40yr	10° ct	2	40yr	Normal vessels, optic disc and macula. Nummular pigment deposits Vitreous floaters	Rods and mix: NR, cones and flicker: reduced amplitude / 40yr	Hyperopia. Progressive hearing loss 36yr
III:4	RP-0030	1971	-	5yr	20yr	38yr	20yr & 38yr	RE = 0.7→0.6LE = 1→0.7	0	20yr	peripheral contraction with temporal islet	1	20yr	typical (few spicules) + hypopigmented lesions and vitreous floaters	Sco and Pho: reduced amplitude of b wave / 20yr. NR / 38yr	Progressive myopia
III:6	RP-0030	1981	10yr	3yr	9yr	-	6yr	RE = 0.7; LE = 1	0	10yr	Ring scotoma	1	-	-	Sco and Pho: reduced amplitude of b wave / 10yr	Progressive myopia
III:2	RP-0711	1967	22yr	14yr	20yr	No at 42yr	30yr & 42yr	RE = 0.85→0.6 LE = 0.7→0.5	0	42yr	10° ct	2	25yr & 40yr	1^st^ evaluation: tapetoretinal degeneration sine pigmenti 2^nd^ evaluation: typical + with chorioretinal degeneration	Rods and mix: reduced amplitude, cones and flicker: normal / 25yr	-
III:4	RP-0711	1973	-	16yr	-	-	38yr	RE = 0.7 LE = 0.5	0	20yr	10° ct	2	38yr	Typical + mild degeneration of macular RPE	-	-
III:5	RP-0711	1979	30yr	-	-	No at 30yr	30yr	BE = 0.8	0	-	-	-	30yr	Normal vessels, and optic disc, few peripheral and perivascular spicules + degeneration of macular RPE	NR /30yr	-
III:6	RP-0711	1954	18yr	Childhood	40yr	54yr	54yr	RE = 0.8 LE = 0.6	0	-	-	-	-	-	NR / 54yr	-
III:7	RP-0711	1952	24yr	20yr	-	45yr	57yr	RE = 0.8	0	57yr	10° ct	2	57yr	(only RE) typical (few spicules, nummular pigment deposits) + degeneration of macular RPE	NR / 57yr	Enucleation of LE in childhood due to glioblastoma. Photopsia
III:8	RP-0711	1958	-	-	-	No at 50yr	50yr	RE = 0.9 LE = 0.8	0	50yr	10° ct	2	-	Typical with conserved optic disc + degeneration of macular RPE	NR / 50yr	-
IV:2	RP-0711	1991	12yr	11yr	11yr	No at 12yr	11yr &18yr	RE = 0.8→0.6 LE = 0.7→0.5	0	11yr	30° ct	1	11yr & 18yr	1^st^ evaluation: tapetoretinal degeneration sine pigmenti Vitreous floaters 2^nd^ evaluation: Normal vessels, optic disc, macula and vitreous. Hypopigmented lesions in mid periphery.	Rods: NR, mix: reduced amplitude of a and b waves, cones and flicker: normal / 12yr. Rods and mix: NR, cones and flicker very reduced amplitude / 18yr	-
II:6	RP-1182	1950	51yr	12yr	-	51yr	54yr	RE = 0.3 LE = 0.6	1	54yr	Central & superior hemifield scotoma	1	58yr	Typical + degeneration of macular RPE and vitreous floaters	NR / 58yr	-
III:8	RP-1182	1976	26yr	26yr	26yr	31yr	31yr	RE = 0.7 LE = 1	0	26	RE = Nasal hemifield scotoma LE = Normal	1	33yr	Normal vessels, optic disc, macula. Nummular pigment deposits in periphery.	Rods, mix and flicker: NR, cones: very reduced amplitude / 33yr	Myopia. Unilateral onset (RE)
II:4	RP-0124	1935	57yr	10yr	10yr	57yr **	57yr → 61yr	RE = 0.1 LE = 0.1	2	57yr	RE 10° ct LE = absolute scotoma	3	57yr	Typical	NR / 57yr	Astigmatism
I:2	RP-0576	1957	38yr	24yr	28yr	52yr	40yr & 52yr	RE = 0.7→0.2 LE = 0.9→0.2	0→1	38yr	10° ct	2	38yr	Typical	Rods, mix and flicker: NR, cones: very reduced amplitude / 38yr. NR / 40yr	-
I:2	RP-1650	-	-	-	-	-	60yr	-	2	-	-	-	-	-	-	-
II:2	RP-1650	1958	42yr	-	-	-	-	-	-	-	-	-	-	-	-	-
III:1	RP-1650	1978	27yr	29yr	25yr	No at 25yr	25yr	BE = 0.6	0	32	<20° ct	2	32yr	Typical	Abnormal / 32yr	-
I:2	RP-1996	1924	-	-	-	-	53yr	(Neither perception nor projection of light)	-	-	-	-	-	-	-	High IOP
II:4	RP-1996	1953	48yr	38yr	48yr	59yr	57yr→59yr	RE = 0.8→0.5; LE = 0.2	1	59	<10° ct	3	59	Degeneration of macular RPE + macular edema + peripheral spicules	Sco, Pho and flicker: reduced amplitude of b wave / 57yr	High IOP
III:3	RP-1996	1980	30yr	20yr	20yr	32yr	32yr	BE = 1	0	30	BE = temporal hemifield scotoma	1	32	Choroidal hypopigmentation (no spicules)	-	FA: choroidal silence with granular alteration and macular pattern / 32yr

Yr: Years. VA: Visual Acuity. VF: Visual Field. Ct: Central. ERG: Electroretinogram. VEP: Visual Evoked Potential. FA: Fluorescein Angiography RPE: retinal pigment epithelium. RE: Right eyes. LE: Left eye. BE = both eyes. Sco = Scotopic. Pho = Photopic.

* All patients with cataracts (except family RP-1996) presented: subcapsular posterior cataracts in BE.

** Patients also presented cortical anterior cataract in BE.

*** VA after cataract surgery.

^#^ The age at onset of cataracts is not available (mature cataract at diagnosis).

^##^ Legal blindness. VA classification^a^: 0 = Normal vision (normal and near normal vision) (≥0.4), 1 = Moderate low vision (<0.4 –>0.1), 2 = Severe low vision (≤0.1–≥0.05, legal blindness), 3 = profound vision loss and blindness (blindness and near blindness, <0.05). VF classification^b^ 0 = normal, 1 = peripheral and ring scotoma, peripheral constriction with VF ≥20°, 2 = <20°–≥10° central, 3 = <10° central. Typical fundus: optic disc pallor, attenuation of the retinal vessels and pigmentary deposits resembling bone spicules. IOP: Intraocular pressure. NR: Non recordable.

In most cases NB was the first noticeable symptom with an onset in pre-adolescence or early twenties (mean age 15.9 ± 10.1 years). Visual field (VF) loss onset was variable, although tubular vision onset tended to occur around the third decade of life (mean age 23.2 ± 11.9 years) and stayed stable until an advanced age. Loss of visual acuity (VA) appeared later in the disease´s evolution, being normal (≥0.4) until the fifth decade of life (Table 2).

**Table 2:** Phenotypic characteristics (means and standard deviation) of patients with the p.Gly56Arg mutation in *NR2E3*.

Age at diagnosis (yr)	NB Onset (yr)	VF loss Onset (yr)	Cataract (yr)	VA		VA (WHO severity)		Fundus		ERG	
								Typical	Macular alteration	Impaired (yr)	Non recordable (yr)
**30.2 ± 14**	**15.9 ± 10.1**	**23.3 ± 11.9**	Yes = 14, No = 5, NA = 5	<50yr **RE = 0.70 ± 0,17; LE = 0.72 ± 0.23**	>50yr **RE = 0.3 ± 0,3; LE = 0.3 ± 0,3;**	<50yr **Normal vision**	>50yr **Moderate to profound vision loss**	**63.2%** (12/19)	**47.4%** (9/19)	**29.2 ± 13.9**	**54.55 ± 14.4**

Yr: Years. VA: Visual Acuity. VF: Visual Field. ERG: Electroretinogram. NA: Not Available. RE: Right Eye. LE: Left Eye. VA classification^a^: 0 = Normal vision (normal and near normal vision) (≥0.4), 1 = Moderate low vision (<0.4 –>0.1), 2 = Severe low vision (≤0.1–≥0.05, legal blindness), 3 = profound vision loss and blindness (blindness and near blindness, <0.05). Typical fundus: optic disc pallor, attenuation of the retinal vessels and pigmentary deposits resembling bone spicules.

Only two patients presented legal blindness (VA ≤0.1 or VF ≤10°) at 60 and 71 years of age, respectively. Additionally, one patient with RP, who also presented glaucoma, was diagnosed with complete blindness (neither perception nor projection of light) at the age of 53.

More than half of the patients (58.3%) with available ophthalmological data presented cataract (73.7%), most of them (50%) since the third decade of life (Table 2).

**Fig 2:**
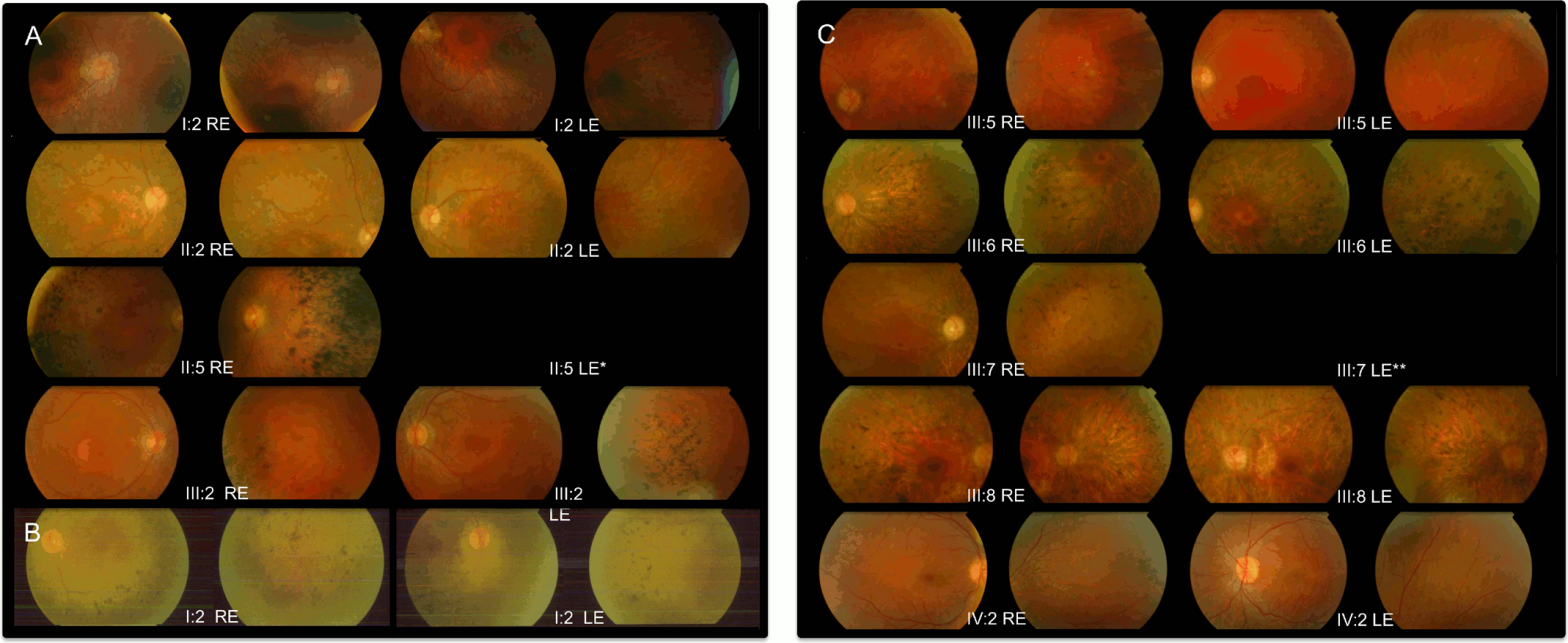
Fundus Images: Each patient presents two fundus images per eye. A) Family RP-0030, B) Family RP-0576, C) Family RP-0711. *Fundus image not available. **Enucleation of the LE due to glioblastoma. RE = Right eye. LE = Left eye.

All ophthalmoscopic examination data available (19 patients) showed fundus alterations (Fig 2).

These fundus alterations could be detected since the beginning of the vision impairment, although visual acuity was not affected (Table 1).

Although fundus changes showed inter and intrafamiliar variability, most of the patients showed typical RP changes with the progression of the disease. It was also frequent to find macular affectation (47.4% -Table 2-) with preserved visual acuity, even when patients were studied in the first stages of the disease (family RP-0711, IV:2 -Table 1-).

ERG recordings were available for 19 cases. All available ERG recordings (scotopic, photopic and flicker) showed alterations from the beginning of the disease, and these changes could be detected around the first decade of life, when studied at that age (family RP-0711 patient IV:2; and family RP-0030 patient III:6—Table 1 and S1 Fig). Non recordable ERG occurred around the age of fifty (Table 2). Similarly as for fundus changes, the initial impairment in ERG recordings did not seem to alter visual acuity.

### Haplotype analysis

**Fig 3:**
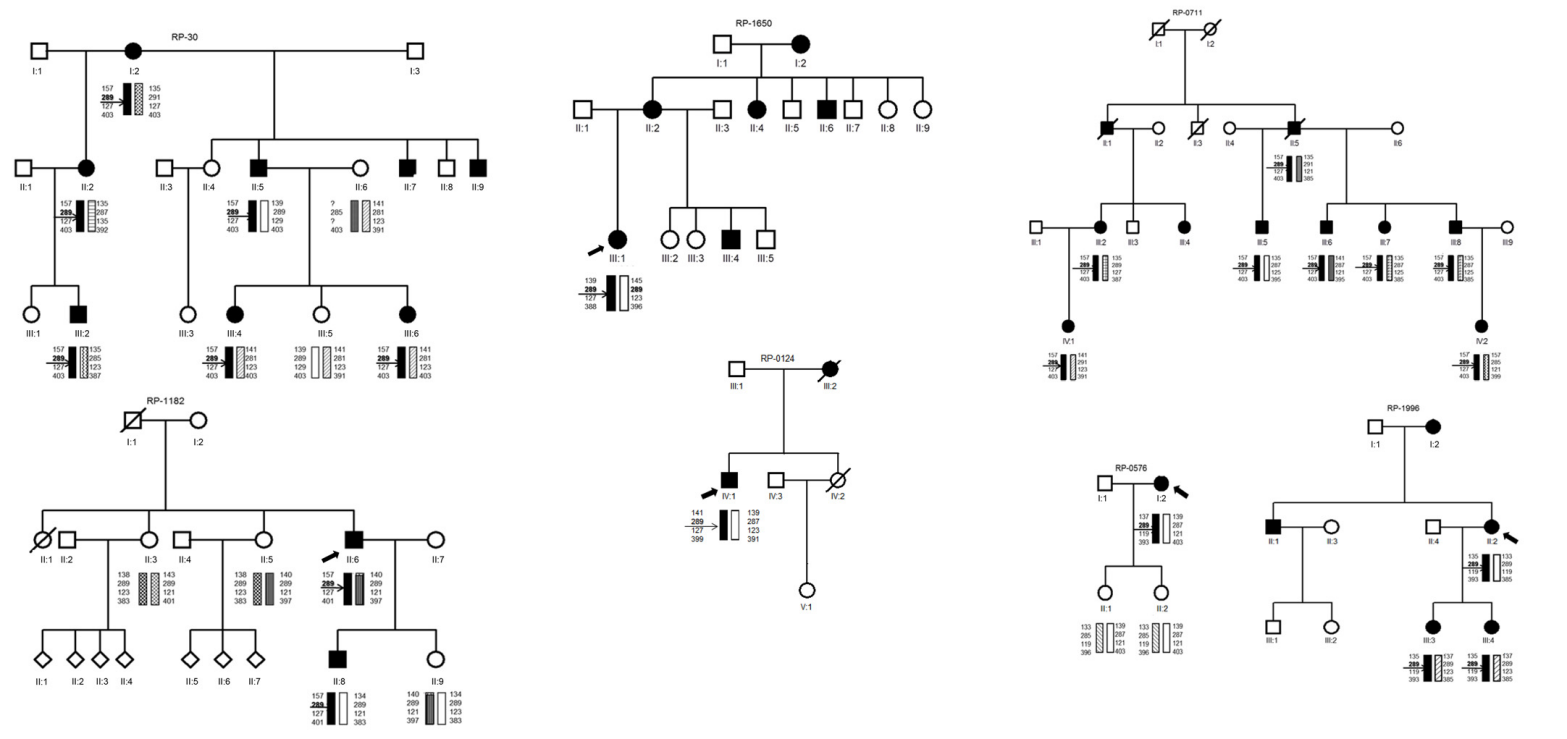
Pedigrees and Haplotype analysis of adRP families with p.Gly56Arg mutation in *NR2E3*. Haplotype analysis: → marks the *NR2E3* gene position. The *NR2E3* extragenic polymorphic markers used are D15S967, D15S1050, D15S204 and D15S188.

Four of the families (RP-0030, RP-0576, RP-0711 and RP-1182) belonged to the same town in Spain (in the Toledo region, with 2755 inhabitants in 1920 and 3511 inhabitants in 2010, according to the demographic data of the Instituto Nacional de Estadística -INE- http://www.ine.es). In two of these families, RP-0030 and RP-1182, a common ancestor (four generations above the respective family probands) could be identified, but not in RP-0711 and RP-0576 (family trees investigated five generations above the respective probands, Fig 3).

Inferring haplotypes in families RP-1650 and RP-0124 (and even in RP-1996) is very speculative. However, the two large families belonging to the same village (RP-0030, RP-0711) shared a common haplotype for the four flanking biomarkers [D15S976 (157bp allele)—D15S1050 (289 bp allele)—D15S204 (127 bp allele)—D15S188 (403 bp allele)] linked to the mutation and RP-1182 shared the same haplotype for the markers D15S976-D15S1050-D15S204. Besides, RP-0124 and RP-1650 families shared a common haplotype defined by the two flanking markers D15S1050 (289bp allele) and D15S204 (127bp allele) whereas the other two families, one from the same city (RP-0576) and RP1996 showed the same proximal marker (D15S1050 289bp) but differed in the distal one (D15S204 119bp allele) indicating that at least two independent mutational events or alternatively a postmutational recombination event, might have occurred (Fig 3).

So, D15S1050 (289bp allele), the closest microsatellite to *NR2E3* gene, is shared by all patients, leaving a small region of 104Kb comprised between D15S1050 (ensemble genomic position Crh15: 71,980,969–71,981,252) and the *NR2E3* gene (ensemble genomic position Crh15: 72,084,977–72,110,600), which could be common to all of the patients carrying p.Gly56Arg mutation.

## Discussion

This paper is an extensive report of frequency of the mutation (p.Gly56Arg) in the *NR2E3* gene in 24 cases diagnosed of adRP, and the associated phenotype.

The prevalence for this mutation in our present cohort of adRP families is 3.5% which is similar to the rates described in Europe (3.4%) [17], but higher than those described in North America [19].

In our cohort of adRP families, the p.Gly56Arg mutation is the second most common single mutation detected [24] after the p.Pro347Leu mutation in the *RHO* gene which was found in nine families out of 200, representing a 4.4% [25].

The results of the haplotype analysis are conclusive for the two most informative families belonging to the same small village (Fig 3). We found a common haplotype in those families for the four markers and for three of them in the family RP-1182. These data support an ancient founder effect for the p.Gly56Arg in our families. However one must be cautious because i) one out of four families coming from the same small geographical area did not share the common haplotype. This fact is unexpected and despite there being an association between the 289 allele of D15S1050 and p.Gly56Arg, D15S1050 and *NR2E3* are in different disequilibrium blocks, we have to consider that the Spanish population is underrepresented in the Hapmap project and that the allele 289 has a frequency of 0.519 (http://www.genoscope.cns.fr/externe/gmap/Nature-1995/), so the probability that both the allele 289 and the mutation are located in the chromosome is very high. Furthermore, there are no hot spot recombination sites like the common motif CCNCCNTNNCCNC [26] within the *NR2E3* gene and their 5’ and 3’ UTR regions that could explain the lack of a common ancestral haplotype by a high recombination rate, ii) The frequency of p.Gly56Arg in populations that have evolutionary diverged, such as American, European and Chinese, are high and quite similar (1.2% in American [19], 3.4% in European [17] and 1.2% in China [27]) and iii) the mutation c.166G>A is a change GGG>AGG in codon 56 of *NR2E3*. This change lies on a CpG dinucleotide and has been reported as high *de novo* mutation site. In fact, the most common *de novo* mutated codon associated with human disease is a GGG>AGG or CGG mutation in codon 380 of *FGFR3* gene [28, 29] which results in achondroplasia. In summary, it is tempting to speculate that the p.Gly56Arg arose once in our population as a very ancient hit giving time for a number of recombination events that have led to the change in the ancient haplotype.

The *NR2E3* gene is associated with both autosomal recessive and dominant retinitis pigmentosa. The *NR2E3* gene recessive mutations present variable phenotypes (ESCS, GFS and CPRD) with variable ophthalmological findings, but all showing night blindness, rudimental or absent rod function, and hyperfunction of the "blue" S-cones [14, 20, 30]. Dominant *NR2E3* gene mutation has been associated with RP phenotype [17–20] and to date, the phenotype has only been described in five European [17, 18, 20] and four North American [19] families characterized by presenting RP, but not to other phenotypes associated to *NR2E3* gene recessive mutations [16, 17]. However, some affected members of those families displayed phenotypic similarities to ESCS [17, 20].

Although we observe, as it has been previously described [18], inter and intrafamiliar phenotype variability for the p.Gly56Arg mutation, some genotype-phenotype correlation appears to be quite apparent, not only homogeneous progression [19], but occurrence of vision impairment milestones. Patients with *NR2E3* dominant mutation showed a moderate form of retinal dystrophy. Thus, patients in our cohort present early NB onset around puberty, preservation of VF>10° until the 4^th^-5^th^ decade of life, normal or moderately low VA (WHO criteria) to an advanced age and preservation of photoreceptor function (recordable ERG) until the 4^th^ decade of life (Table 2).

It is important to notice that this phenotype is milder than the one found in recessive forms [18] and to point that the fundus alterations (additionally to typical RP fundus, atypical findings are present as macular changes and choriocapillaris atrophy) and ERG findings (impaired photopic and flicker records in the first stages of the disease) observed in some of our patients, are not the classical RP changes previously described in the European and North American *NR2E3* dominant patients [17, 19, 20].

Moreover these changes did not seem to correlate with visual acuity impairment, at least during the first stages of the disease (impaired ERG and fundus changes with normal VA). However the absence of correlation between ERG and VA is common in RP patients [1].

These fundus changes have recently been described on the ophthalmoscopic findings of *NR2E3* ESCS (recessive) patients. These changes can lead to a misdiagnosis if family history, initial symptoms reported by patients and progression of the disease in the early stages are not queried when performing the clinical history. Besides, other ophthalmological signs such as double concentric autofluorescence ring (not performed in our cohort), that has been described as an initial sign of retinal degeneration in patients carrying the *NR2E3* dominant mutation [20], could help in the molecular diagnostic orientation of adRP patients.

Furthermore, some of these changes such as macular edema, can, at least partially, be treated [31, 32], which is important for prognosis, disease outcome and follow up of these patients. Although this event was only observed in one of our patients by ophthalmoscopic examination, we cannot discard that it could be present in other NR2E3 adRP patients, as no OCT data was available for the present cohort.

## Conclusion

We believe that the relevance of this study is not only being the single largest *NR2E3* genotype-phenotype correlation study performed to date, but also highlighting the importance of p.Gly56Arg in the *NR2E3* gene associated to adRP.

*NR2E3* is responsible for two main retinal phenotypes ESCS (including Goldmann-Favre syndrome) only for recessive forms and RP (including CPRD) related to both dominant and recessive forms. However, in our cohort there is a wide range of phenotypic characteristics that differ from typical RP phenotype and resemble other *NR2E3* phenotypes such as ESCS and CPRD, mainly at a fundus level where we found macular disturbance, which is frequently seen in ESCS, in 50% of our evaluated patients and two patients with nummular pigmentation (typical of CPRD phenotype).

In this report, we are providing new clues of a characteristic phenotype for this mutation that allows for making an estimated prognosis of autosomal dominant RP due to p.Gly56Arg mutation in the *NR2E3* gene. Besides, we believe that this study can improve molecular diagnosis approach, clinical management, risk assessment, and genetic counselling of adRP patients and their families.

## Supporting Information

S1 Fig Electroretinogram (ERG) recording in IV:2 member of RP-0711 family. A) ERG at 11years of age. B) ERG at 18 years of age.
(DOCX)
