# Supplementary material for: Dominant Retinitis Pigmentosa, p.Gly56Arg Mutation in NR2E3: Phenotype in a Large Cohort of 24 Cases
Source: PLoS One. 2016 Feb 24;11(2):e0149473. doi: 10.1371/journal.pone.0149473 (PMC4766102; doi:10.1371/journal.pone.0149473)
Supplement: S1 Fig — A) ERG at 11years of age. B) ERG at 18 years of age. (DOCX) [file pone.0149473.s001.docx]

**Supplementary Figure 1:** Electroretinogram (ERG) recording in IV:2 member of RP711 family.

**A**: ERG at 11years**. B**: ERG at 18 years.

**A**

**B**


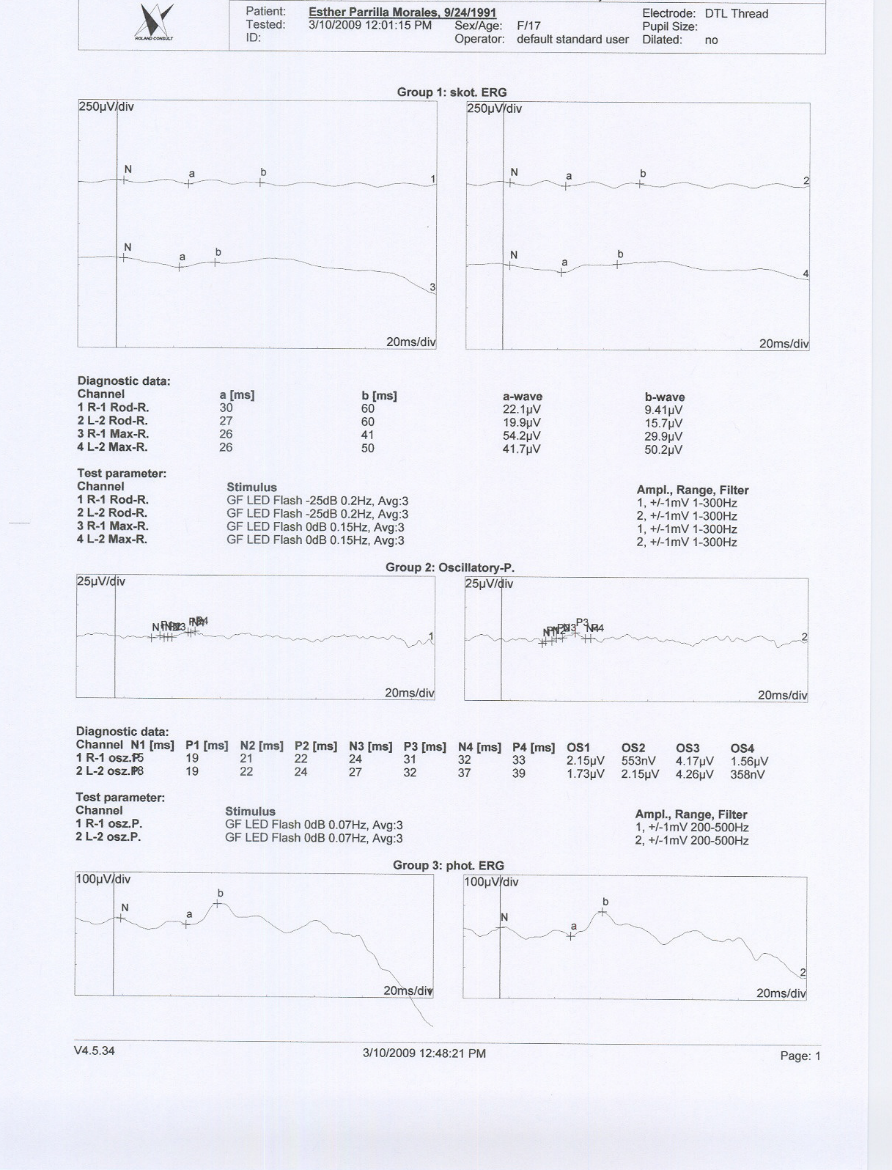

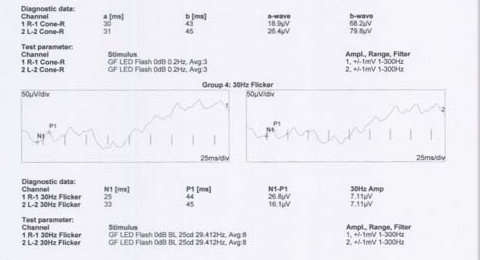

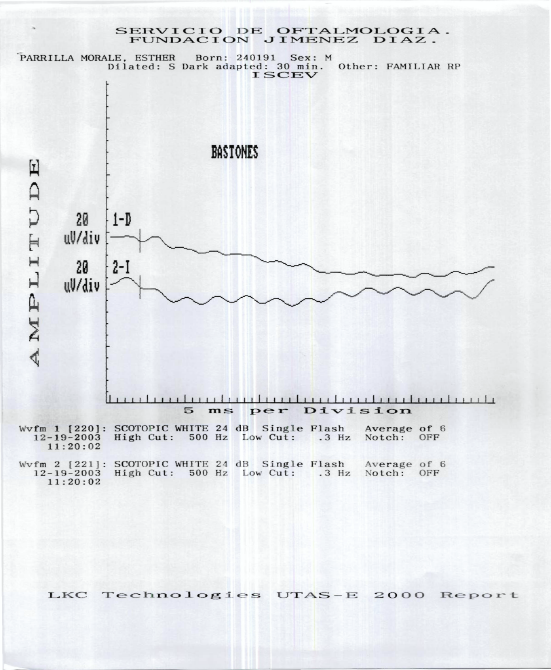

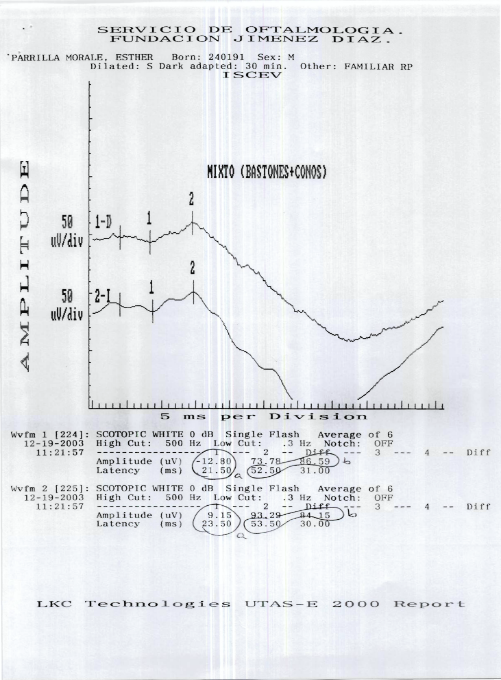

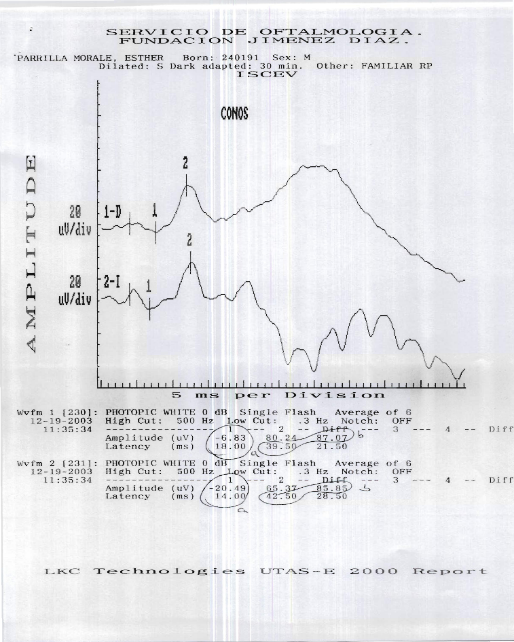

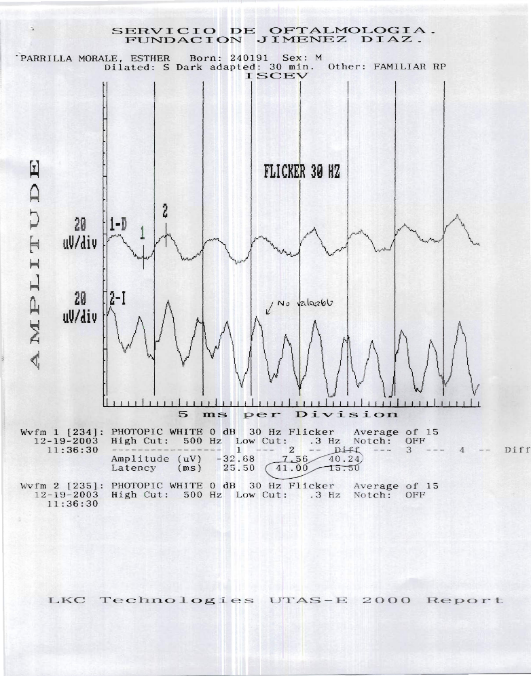


rods

cones

Right eye

Right eye

Right eye

Right eye

Left eye

Left eye

Left eye

Left eye


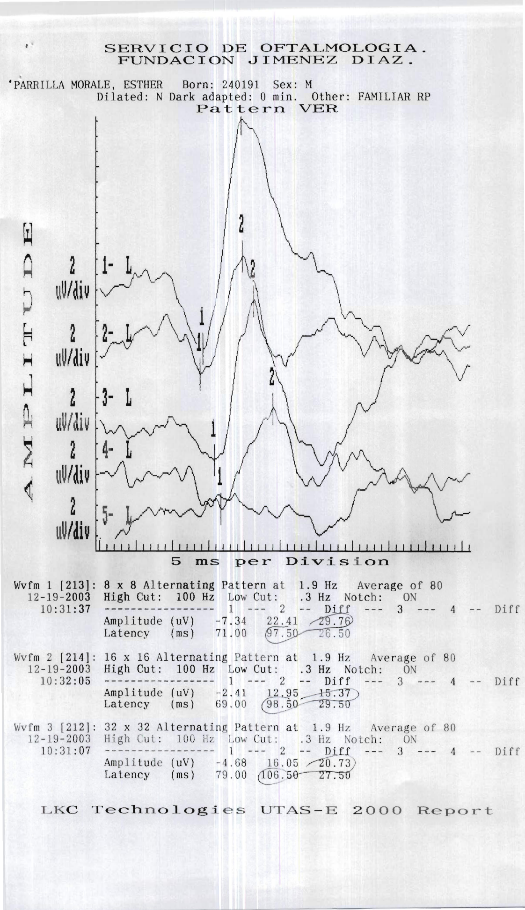

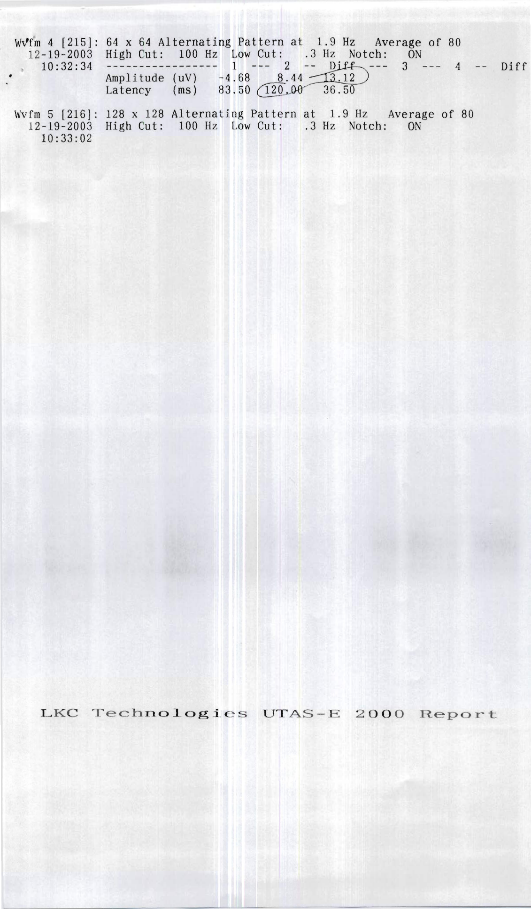


Left eye


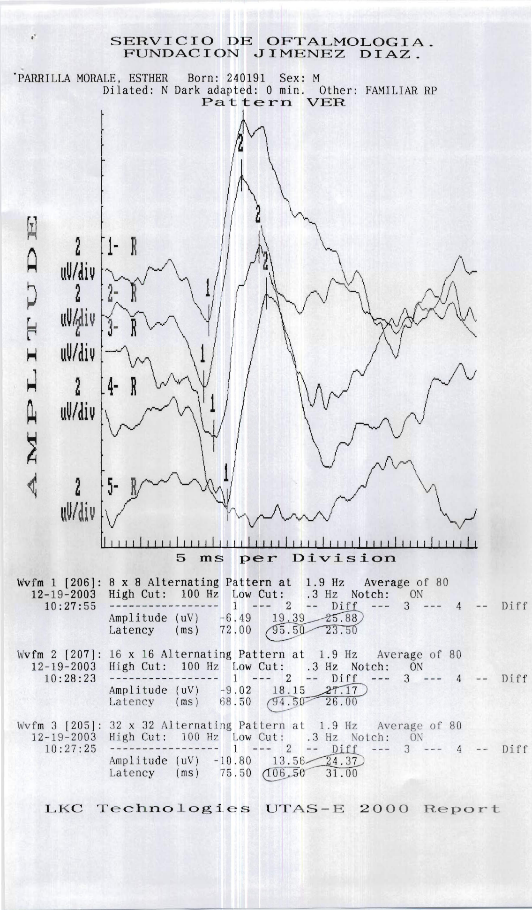

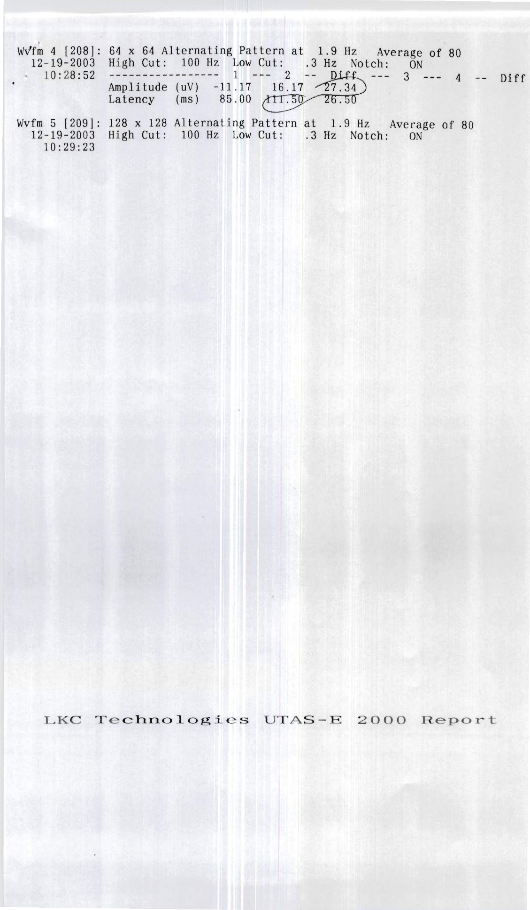


Right eye
